# Proceedings of the first workshop on Peripheral Machine Interfaces: going beyond traditional surface electromyography

**DOI:** 10.3389/fnbot.2014.00022

**Published:** 2014-08-15

**Authors:** Claudio Castellini, Panagiotis Artemiadis, Michael Wininger, Arash Ajoudani, Merkur Alimusaj, Antonio Bicchi, Barbara Caputo, William Craelius, Strahinja Dosen, Kevin Englehart, Dario Farina, Arjan Gijsberts, Sasha B. Godfrey, Levi Hargrove, Mark Ison, Todd Kuiken, Marko Marković, Patrick M. Pilarski, Rüdiger Rupp, Erik Scheme

**Affiliations:** ^1^Robotics and Mechatronics Center, German Aerospace CenterOberpfaffenhofen, Germany; ^2^Department of Mechanical and Aerospace Engineering, Arizona State UniversityTempe, AZ, USA; ^3^Prosthetics and Orthotics Program, Rehabilitation Computronics Laboratory, University of HartfordWest Hartford, CT, USA; ^4^VA Cooperative Studies Program, Department of Veterans AffairsWest Haven, CT, USA; ^5^Department of Advanced Robotics, Istituto Italiano di TecnologiaGenoa, Italy; ^6^The Centro di Ricerca “E. Piaggio,” Università di PisaPisa, Italy; ^7^Department of Orthopaedic Surgery, Heidelberg University HospitalHeidelberg, Germany; ^8^Department of Computer, Control, and Management Engineering, University of Rome La SapienzaRome, Italy; ^9^Idiap Research InstituteMartigny, Switzerland; ^10^Department of Biomedical Engineering, Rutgers UniversityPiscataway, NJ, USA; ^11^Department of Neurorehabilitation Engineering, University Medical Center, Georg-August-UniversityGoettingen, Germany; ^12^Institute of Biomedical Engineering, University of New BrunswickFredericton, NB, Canada; ^13^Rehabilitation Institute of Chicago, Northwestern UniversityChicago, IL, USA; ^14^Department of Computing Science, University of AlbertaEdmonton, AB, Canada

**Keywords:** human–machine interfaces, prosthetics, rehabilitation robotics, EMG, prosthetic control

## Abstract

One of the hottest topics in rehabilitation robotics is that of proper control of prosthetic devices. Despite decades of research, the state of the art is dramatically behind the expectations. To shed light on this issue, in June, 2013 the first international workshop on *Present and future of non-invasive peripheral nervous system* (*PNS*)*–Machine Interfaces* (MI; PMI) was convened, hosted by the International Conference on Rehabilitation Robotics. The keyword *PMI* has been selected to denote human–machine interfaces targeted at the limb-deficient, mainly upper-limb amputees, dealing with signals gathered from the PNS in a non-invasive way, that is, from the surface of the residuum. The workshop was intended to provide an overview of the state of the art and future perspectives of such interfaces; this paper represents is a collection of opinions expressed by each and every researcher/group involved in it.

## INTRODUCTION AND MOTIVATION

The first international workshop on *Present and future of non-invasive PNS*–*Machine Interfaces* took place in June, 2013 in Seattle, USA, hosted by the 13th International Conference on Rehabilitation Robotics (ICORR). The keyword *peripheral nervous system (PNS)*–*Machine Interface* (MI; PMI from now on) was chosen to denote one of the hottest topics in the rehabilitation robotics community, namely the interpretation of biological signals extracted non-invasively from the PNS, with the intent to equip an individual with disability to *reliably*, *dexterously,* and *naturally* control a robotic artifact gifted with many degrees of freedom (DOFs).

In the paradigmatic case, surface electromyography (sEMG) is used as the main source of signals, and the complexity of modern upper-limb prostheses (self-powered mechanical shoulders, elbows, wrists, hands, and fingers) represents a formidable challenge and an ideal benchmark for the PMI dealing with the problem. sEMG has been in use since the 1960s to proportionally control single-DOFs hand grippers since it involves neither surgery nor hospitalization, its signal remains rich in information even decades after an amputation, and it provides clearer signals than brain–computer interfaces based upon, e.g., electroencephalography. The application of machine learning to sEMG has been proposed since the 1960s as a means of converting electrical activation signals to useful control signal for arm and hand prostheses; nevertheless, the state of the art of control is still poor.

Literally dozens of different approaches have been applied to sEMG to decode an amputee’s intentions, but none has as yet made it to the clinics: as a PMI, sEMG has revealed to be unreliable, badly conditioned, subject to change with time, fatigue, and sweat. No valid alternatives to sEMG are used in the clinics, whereas dexterous prosthetic artifacts are now appearing on the market, demanding ever better control by the patient.

The workshop revolved around four “themes” or “questions,” with the aim of shedding at least a partial light on some of them:

(1) what is wrong with sEMG? why do clinicians not use it?
(2) how can sEMG be better used?
(3) what alternative, radically new solutions are available, if any?
(4) what are the benefits of sharing control between the human subject and the prosthesis?

Ten invited talks were given at the workshop, in which each research group gave a broad overview of its activities and offered its point of view on one of the above topics. This paper collects the opinions appeared in the workshop.

The remainder of the paper is organized according to the four above questions; an overview of the talks, as well as a presentation of the workshop and of the PNS–MI workgroup, can be found at the URI pnsinterfaces.wordpress.com.

## WHAT IS WRONG WITH EMG? WHY DO CLINICIANS NOT USE IT?

Merkur Alimusaj, Levi Hargrove, Todd Kuiken, and Rüdiger Rupp.

### WHAT IS WRONG WITH IT?

The benefit and acceptance of myoelectric prostheses are influenced by a number of reasons: weight, noise, cosmetic appearance, battery duration, price, and expense of servicing. State-of-the-art mechatronic devices (prosthetic hands, wrists, elbows, etc.) aim to increase the number of motorized DOFs available. To make full use of these multifunctional prosthetic systems an appropriate user interface must be implemented. Several research groups are working in the field of sEMG pattern recognition, trying to create a more robust prosthetic control by adding predictors into control schemes. Although a lot of work has been done, only marginal progress has been made in the clinically available solutions for prosthetic control. A bigger effort is therefore needed to address the prosthesis user’s needs within multidisciplinary projects. This leads to the necessity of putting the user at the center of the research and shaping research to target clinical relevant outcomes.

### THE GRAND GOAL: TOTAL RESTORATION

Loss of a hand or an arm due to an amputation dramatically decreases the quality of life. The amputee has not only lost her/his grasping functions, but also an important communication tool. Not only amputees, but also patients with congenital deformities are prosthetic users and should therefore be addressed. Since the human hand/arm has more than 20 DOFs, the idea of a complete substitution by a prosthesis represents a highly ambitious goal.

**FIGURE 1:**
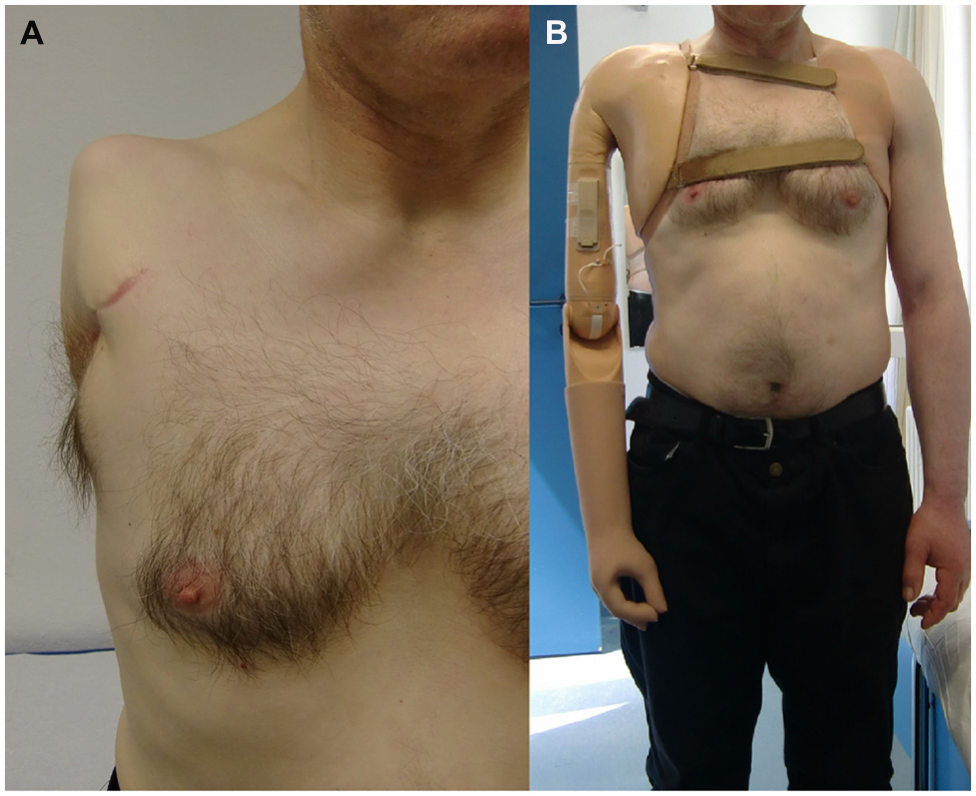
**A myoelectric prosthesis implanted after shoulder disarticulation **(A)** 2-DOFs self-powered hand, wrist, and elbow, plus non-motorized mechanical shoulder with electrical fixation **(B)****.

The dexterity of current prosthetic effectors is not yet anywhere near that of the human upper limb. The lack of functionality and intuitive control increases by the level of amputation – most dramatically at the level of shoulder disarticulation or four-quarter amputation; in such cases the usage and acceptance of currently available upper extremity prostheses is dramatically low (Peerdeman et al., 2011; Østlie et al., 2012), mainly due to the lack of sensory signals to deal with, and with the weight imbalance caused by the harness and the devices themselves. **Figure 1** shows the typical harness implanted on a patient of shoulder disarticulation.

The replacement of a human hand by means of a prosthesis already poses a number of challenges from the mechatronic point of view. However, the control issue might be even harder (Peerdeman et al., 2011). This leads to the conclusion that an increased number of DOFs of prosthetic components is only the first step in improving these systems. Limitations in articulated control become even more apparent in the case of multi-fingered prosthetic hands, which are still controlled by a conventional two-sEMG-electrode configuration. Such prostheses are operated via a non-physiological and non-intuitive series of muscle co-contractions. This control strategy results in a rather slow transfer of the user’s intentions to an action by the prosthesis and needs a relevant training period assisted by highly educated experts. The limitation of the control interface does not allow for full exploitation of the mechanical dexterity of current multi-fingered hands. Taking into account that this control strategy is also used in higher amputation levels like transhumeral amputations or shoulder disarticulations, it is obvious that the mental effort and workload of a user exponentially increases with level of impairment. The user needs to control at least three independent components (hand, wrist, and elbow) and co-contractions are needed to switch between operation of each component. Simultaneous and proportional activation of all DOFs or components is, at the time of writing, still a dream.

### STATE OF THE ART

Targeted muscle reinnervation (TMR) transfers the residual motor branches of arm nerves to alternative muscle sites (Kuiken, 2006), while sensory nerves are surgically reconstructed in shoulder disarticulated patients at the skin near the neck for tactile feedback. If TMR is successful, it projects the muscles of the hand to chest muscles, thereby increasing the number of sites available for recording specific sEMG signals for prosthetic control. Currently, data from up to six sEMG electrodes are considered without the need for any sEMG pattern recognition algorithms. Using pattern recognition methods in combination with TMR, prosthesis with a more intuitive and robust control were developed and applied in patients (Kuiken et al., 2009). TMR is now a procedure that is performed clinically at institutions around the world.

TMR was developed primarily to create independent EMG control sites for proximal level upper-limb amputees. TMR is also suitable for other amputations levels. For example, analysis of high-density surface EMG signals shows that information corresponding to intrinsic hand-muscles may be decoded using pattern recognition (Zhou et al., 2007). Our clinical observation is that TMR amputees can control multiple hand grasps easier and more reliably than transradial amputees supporting the application of TMR to this population (Li et al., 2010). We have also shown that TMR has applications for lower-limb amputees using powered prostheses. It improves control during ambulation and allows the amputees to independently reposition their knee or ankle to prepare for difficult transfers (Hargrove et al., 2013b). Finally, there is compelling data to suggest that TMR is an excellent treatment for neuroma pain and likely prevents neuroma formation. After nerve transection in an amputation, the proximal nerve attempts regeneration with significant sprouting at the nerve stump terminus (Zhang and Fischer, 2002). If the nerve is unable to reconnect to a target, sprouting may progress to form a neuroma: a dense, poorly organized mass of neurons in connective tissue (Song et al., 2006). In TMR, the amputated nerve is sutured to the motor point of a nerve that previously innervated the TMR target muscle. In a rabbit model, transferring brachial plexus nerves to denervated muscle reduced axonal sprouting by over 50% and reduced neuroma size (Kim et al., 2012; Ko et al., 2013). In a retrospective review of TMR patients with preoperatively painful neuromas, 14 of 15 patients had complete resolution of their pain, and the remaining patient had a significant reduction in neuroma pain (Ko et al., 2013), such that he could wear a prosthesis.

Simultaneous and proportional control of more DOFs has highest priority in research and clinical routine (Jiang et al., 2012a). According to this schema, patients are able to control, e.g., wrist and hand motion without the need of learning artificial co-contraction sEMG patterns to switch between the control of prosthetic components. With an increasing number of EMG signals, the control becomes more intuitive and robust, even with the use of non-invasive sEMG electrodes (Hargrove et al., 2013a). Nevertheless, prosthetic control via sEMG is inherently influenced by different disturbances such as, e.g., muscular fatigue, signal degradation due to sweating, inadequate positioning of the socket, stump volume fluctuation, and cognitive effort. This leads to high variations in the user’s ability to ensure a safe and stable prosthetic control in particular over several hours. Incorrect operation of the prosthesis raises the level of frustration and herewith the tendency of rejection of the device.

In addition to the developments in pattern recognition and surgical intervention, relevant effort has been spent on the improvement of motion prediction (Pilarski et al., 2013b), i.e., analysis of sEMG patterns to predict the arm kinematics. An incorporation of predictors into prosthetic control schemes could lead to a reduction of latency in prosthetic action. Implementing a robust method within the control algorithm for prediction of the user’s intention shall improve the “speed” of control, the functional outcome and the user’s satisfaction with the device.

### CHALLENGES FOR THE CLINICIANS

**FIGURE 2:**
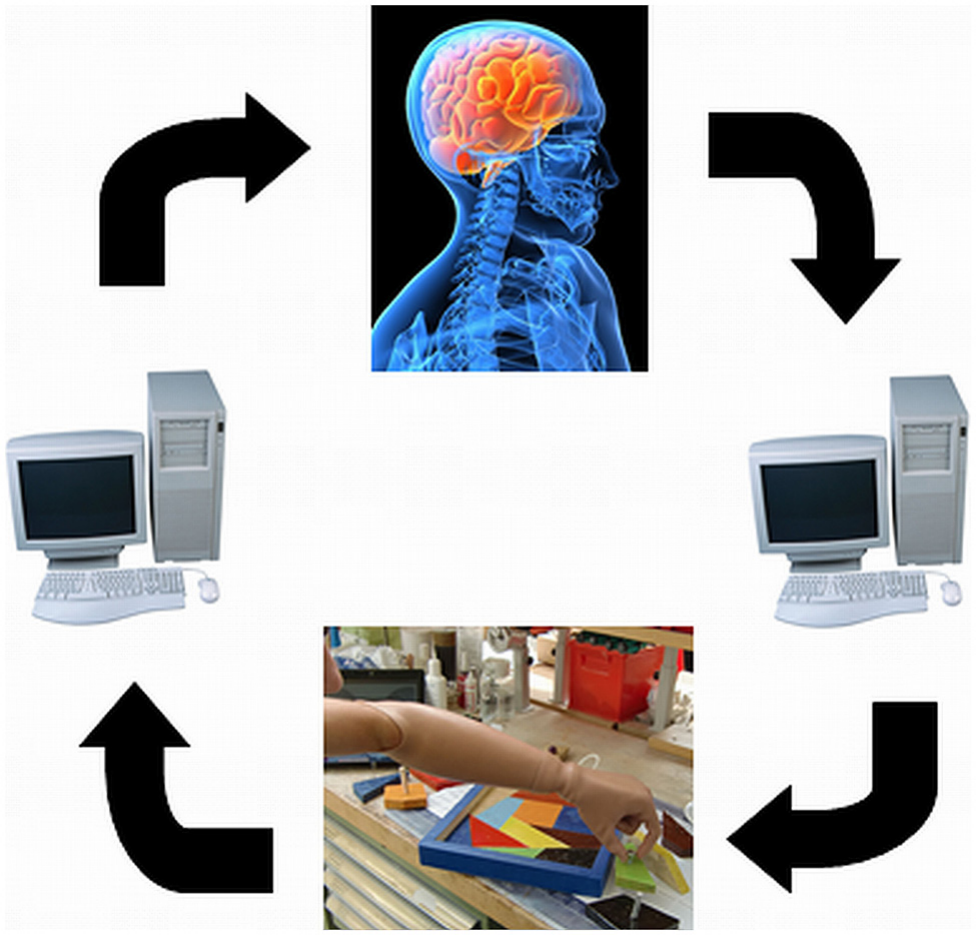
**Closed-loop prosthetic control: including appropriate feedback for an increased embodiment**.

One of the major limitations of current devices is the lack of feedback to the user about forces or position of the prosthesis. To achieve an intuitive and reliable control, feedback must be provided (Dhillon and Horch, 2005). Feedback will support the embodiment of the whole device consisting of the prosthetic socket and the components of the prosthesis itself (**Figure 2**).

Most of the research in the field on advanced prostheses is currently carried out in robotics/electrical engineering laboratories. Like in other fields of assistive technology, the introduction of an iterative user-centered design is needed to establish a close link between researchers, clinical experts, professional, and end-users. The feedback of end users on the usability of complex prostheses developed by engineers and robotic specialists would help to come up with devices that match the user needs and capabilities; clinical evidence shows that the high variability in the population of amputees needs individual solutions, not only at the level of mechatronic components but also for the socket, the control systems, and the training. Prosthetic fitting is always dependent on the patient’s needs and individual anatomy which all should be addressed by including clinical aspects in the development process of novel devices.

Future work should target the integration of additional and/or novel sensors and sEMG arrays (Youn and Kim, 2010; Cooper et al., 2014) within the prosthetic socket. Embedded sEMG sensors in the socket could also lead to more robust control. Invasive, minimally invasive, and non-invasive methods should be targeted. Furthermore, electrocutaneous or vibrotactile stimulation as a feedback system for the first contact to an object, for slip detection, and of the grasp force, could lead to better embodiment of the prosthesis (Peerdeman et al., 2011).

## HOW CAN sEMG BE BETTER USED?

Barbara Caputo, Kevin Englehart, Arjan Gijsberts, Patrick M. Pilarski, and Eric Scheme.

### OVERVIEW

**FIGURE 3:**
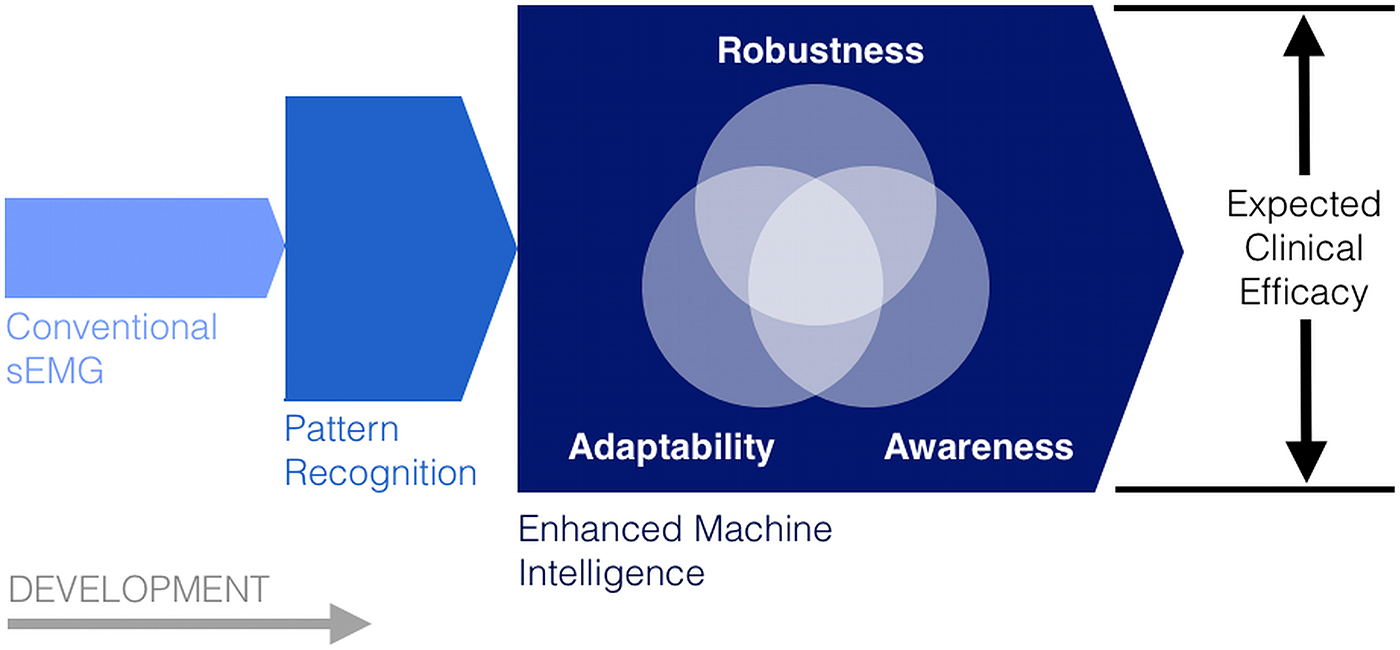
**Robustness, adaptability, and situational awareness (sensorimotor knowledge) as three complementary machine intelligence pursuits to enhance the expected clinical effectiveness of conventional and emerging myoelectric control systems**.

In light of the observed limitations to conventional myoelectric control, a number of approaches have been developed to more effectively process and use existing myoelectric control information. In particular, enhanced pattern recognition and other forms of machine intelligence have been recently deployed to increase the *robustness*, *adaptability*, and *situational awareness* of myoelectric control systems and other human–machine interfaces (**Figure 3**). As a whole, the studies reviewed in this section suggest that, by increasing the decision making and information processing capacity of sEMG control technologies, it may soon be possible to surpass many of the existing barriers to their use in a clinical setting.

### ENHANCING THE ROBUSTNESS OF PATTERN-RECOGNITION-BASED MYOELECTRIC CONTROL

Pattern-recognition-based myoelectric control has been discussed in the research literature for decades (Parker et al., 2006; Oskoei, 2007) but has only very recently been deployed commercially. Consequently, recent work has focused on identifying the reasons that have inhibited its successful transition into clinical practice. Various arguments have been made; however, most have pointed to concerns about clinical robustness (Scheme and Englehart, 2011). Transitioning away from conventional laboratory testing poses significant challenges as many confounding factors are introduced during clinical use. It has been proposed that increases in signal variability during functional tasks contribute to degradation in repeatability, and as a result, overall performance. Hargrove et al. (2006) showed deterioration of performance due to electrode shift, but suggested that it could be minimized by pooling data from shifted electrodes during training. Young et al. (2011) found similar results relating to electrode size, spacing, and orientation. Scheme et al. (2010, 2011) showed the negative effect of changes in residual limb position. Since then, several groups have reiterated these results, concluding that the inclusion of multiple limb positions during training can minimize these effects (Fougner et al., 2011; Geng et al., 2012; Jiang et al., 2012b). Lorrain et al. (2011) investigated the importance of the training set when testing with dynamically varying data. Similarly, Scheme and Englehart (2013a) examined the consequence of using proportional control concurrently with pattern recognition on classification accuracy. Both groups found that training with dynamically varying data helped to drastically improve the robustness of the control scheme.

Each of these studies indicated a need for a more comprehensive representation of the usage case during training. This suggests that the common approach to classification validation in the literature (constrained, moderate intensity contractions) yields only a sparse population of the discriminatory feature space. This sparsity, typically combined with highly repeatable experimental conditions, has led to the observation that the selection of classifier has a minimal effect on the overall system performance (Hargrove et al., 2007). Incorporation of multiple sources of variability during training data collection, however, can be burdensome on the users and clinicians. As more of these factors are identified, this may become a prohibitively intensive approach. Ultimately, it is not reasonable to represent all possible variations during training, inevitably resulting in patterns being elicited during functional use that were unaccounted for during training.

Another criticism of pattern-recognition-based approaches has been a perceived lack of visibility into its inner workings (Lock et al., 2011). Some groups are working to improve understanding of the training process (Powell and Thakor, 2013), but the basic premise of pattern recognition is unchanged. The assumption that a user will only elicit patterns associated with one of *n* motions is predisposed to fail as more challenging usage scenarios are introduced and more sources of variability are added. Instead, the myoelectric control task may more naturally lend itself to a detection problem, where the presence of a known/desired signal is not guaranteed. This subtle difference in philosophy accommodates the notion that observed active myoelectric signals may not result from an intention to activate the prosthesis – current pattern-recognition-based systems, however, do not consider this scenario; rather, the assumption is made that all active contractions originate from a desire to activate the prosthesis, resulting in inadvertent movement of the device during aberrant, accidental, or stabilization contractions. Further complicating the matter is the discrete nature of pattern classification, which gives no indication that patterns are changing until an error actually occurs. This greatly limits the ability to anticipate changes in the system and to measure the influence of confounding factors.

Recently, an extension of the commonly used linear discriminant classifier (LDA) was introduced that converted its nonlinear probability outputs into usable confidence scores (Scheme et al., 2013). These confidence scores, bounded between 0 and 1, were used to represent the certainty that a given decision was correct. It has been suggested that inadvertent activation of a device is one of the leading causes of frustration during clinical testing of pattern recognition systems (Hargrove et al., 2010). Assuming no other result such as dropping or crushing an object being held, a user must – at minimum – correct such an error by eliciting a compensatory antagonist motion. Drawing on inspiration from biometrics, Scheme et al. (2013) only actuated motion when the corresponding confidence was above a minimum threshold. Otherwise, the decision was rejected and overwritten with an inactive or *no movement* decision. The introduction of this rejection option complicates the oﬄine quantification of performance because the effect of the tradeoff between false activations and excessive rejection is unclear. Instead, using a real-time Fitts’ law style virtual target achievement test (Scheme and Englehart, 2013b), a significant improvement was seen in *throughput*, *path efficiency*, *overshoot*, *stopping distance*, and *completion rate*. This approach demonstrated the potential for using confidence based rejection to improve performance and robustness by accounting for situations that might fall outside of the naïve assumptions of standard oﬄine classification.

While their work (Scheme and Englehart, 2013a) focused on the realizable improvement through the use of a rejection scheme, it also established a framework for using a classifier’s probabilistic outputs for something more than a discrete class decision. It is clear that the treatment of pattern-recognition-based myoelectric control as a standalone classification task is insufficient. These recent advances suggest that significant gains in robustness may result from a greater emphasis on its use as part of a complete dynamic control system.

### LEARNING TO ADAPTIVELY CONTROL DEXTEROUS PNS–MI DEVICES

One of the main goals of the biorobotics community is to develop hardware and software tools for providing amputees with dexterous, easy to control prosthetic hands. Still, as of today we live a dichotomy between the hardware and software capabilities of such devices. While today’s hardware for robotic hands has reached impressive levels, control over a satisfactory range of hand postures and forces is still coarse. Progress in the field has often been slowed down by the lack of public data collections. Until 2012, only a limited set of data for hand prosthetics was available. Mostly, such proprietary databases contained up to 10 different grasping actions, static hand postures or fingers and wrist movements.

Recently, the first version of the *NinaPro* database (www.idiap.ch/project/ninapro, Atzori et al., 2012) was introduced to the community. This public dataset provides kinematic and sEMG signals from 52 finger, hand, and wrist movements. As such, it supports experiments at a far larger scale than previously used data, challenging machine learning researchers in terms of classification accuracy, dexterity and life-long learning control of PNS–MIs.

Besides dexterity, the problem of hand prosthetic control involves the training time needed by a user to alleviate the inconsistencies between the desired and performed movements. This process can take up to several days and it is generally perceived as very tiring, sometimes painful. As a consequence, amputees often give up and settle eventually for a cosmetic hand. This issue calls for machine learning techniques able to boost the learning process of each user. Adaptive methods (Chattopadhyay et al., 2011; Matsubara et al., 2011; Tommasi et al., 2013), i.e., methods able to exploit knowledge gathered from previous experience to accelerate learning by a new subject—are suitable for this task. Indeed, the experience gained over several source subjects can be leveraged to reduce the training time of a new target user. In this way the learning process does not start every time from scratch, but it reduces to a faster refinement of prior knowledge.

One general issue pointed out by previous work is the time- and user-dependent nature of the sEMG signals (Sensinger et al., 2009; Matsubara et al., 2011). The first is mainly due to fatigue or electrode displacement, while causes of the second are the personal quantity of sub-cutaneous fat, skin impedance, and differences in muscle synergies. Variations among the probability distribution of sEMG signals across different subjects make the experience gained on one person not naively re-usable (Castellini et al., 2009). When designing a prosthetic hand, this problem induces a strong limitation: each user needs a long training time before being able to fully exploit the prosthesis. Adaptive learning methods focus on transferring information between a source and a target domain despite the existence of a distribution mismatch among them (Ben-David et al., 2010; Pan and Yang, 2010). Thus the knowledge originally acquired on the source can be re-used for the target with a benefit that is as more evident as the target training available data is scarce. This fits perfectly with the problem of prosthetics hand control. Consider the ideal case where an amputee wears his new prosthetic hand for the first time and becomes proficient in using it after only few basic exercises. This would dramatically reduce the number of cumbersome training sessions and make the user much more comfortable, leading to a drastic reduction in functional prosthesis abandonment. To reach this goal, the prosthetic hand should be endowed with an adaptive system that is already informed about the possible basic hand movements and refines this source knowledge through few signals collected from the specific target user.

Still, adaptive techniques have been applied only marginally on this problem. In Matsubara et al. (2011), the authors suggest extracting from the sEMG data a user-independent component that can be transferred across subjects. The source and target data coming from different persons can also be combined together after re-weighting as proposed by Chattopadhyay et al. (2011). In Tommasi et al. (2013), the transfer process is formulated as a max-margin learning method and relies on pre-trained models. All these algorithms have been tested on proprietary data of limited dimension, with respect to the number of subject and the number of hand postures considered. Thus, it is not clear how their performance compares against each other, nor how they would perform on the more realistic scenario of larger numbers of subjects and postures. Even more unclear is how the several algorithms proposed so far in the machine learning literature for adaptive learning would perform in the PNS–MI devices domain, and what specific characteristics such algorithms must provide in order to enable natural and stable control of non-invasive prostheses over extensive periods of time.

### LEARNING AND USING SITUATIONAL KNOWLEDGE IN THE CONTROL OF PNS–MI DEVICES

The intuitive sEMG control of multiple actuators and the robust unsupervised adaptation of devices to changes encountered during deployed operation remain important challenges for users of prostheses and other robotic rehabilitation devices. As discussed above, recent advances in machine intelligence and pattern recognition are helping to alleviate some of these challenges by opening up a wealth of improved control options for the users of sEMG-based prostheses (e.g., Sensinger et al., 2009; Micera et al., 2010; Scheme and Englehart, 2011, 2013b; Tommasi et al., 2013). One emerging area of potential benefit is that of *real-time machine learning*, wherein prediction and control information is learned during ongoing operation of a robotic PMI device (Pilarski et al., 2013a). This sub-section therefore discusses work-to-date and future perspectives on the use of real-time sensorimotor knowledge acquisition as a strategy to gain more functionality from both existing and emerging sEMG control solutions.

**FIGURE 4:**
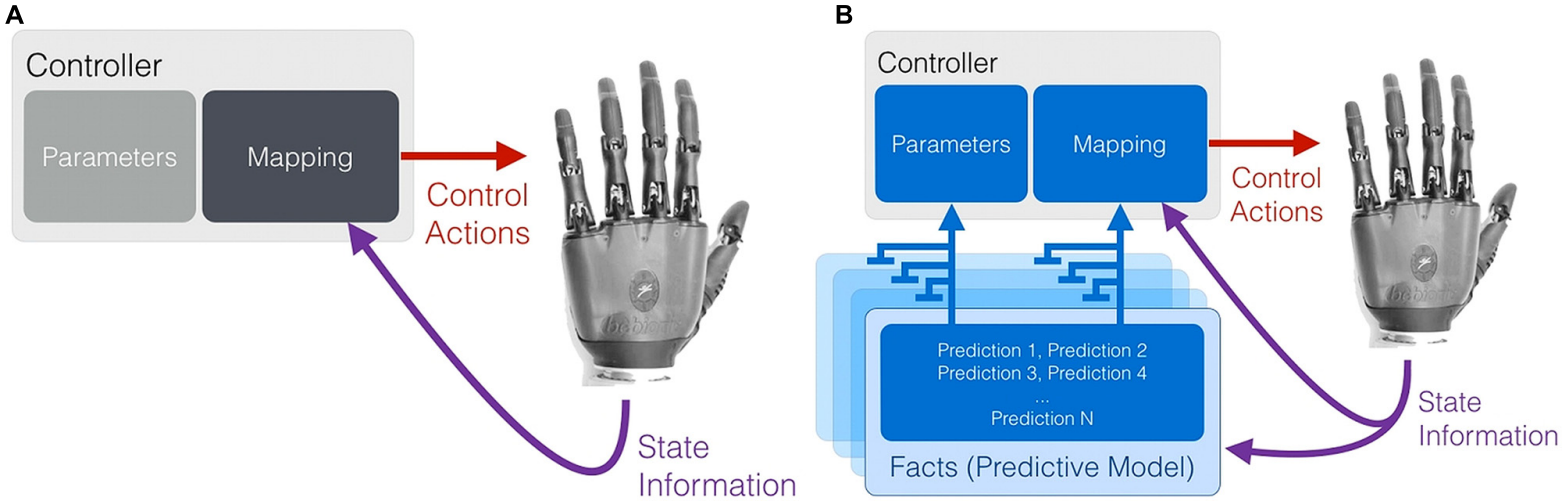
**An abstract representation of the use of situational awareness (knowledge) to supplement myoelectric control.** In conventional myoelectric control, state information in the form of sEMG features is provided to the controller **(A)**. Learned, prediction-based knowledge regarding the context (or contexts) of use can be used to modulate the parameters and the state-action mapping of a controller in a situation- and user-appropriate way **(B)**.

A key point underpinning work on real-time machine learning for PMI control is that contextual or situational awareness (knowledge in the form of temporally extended predictions) is important for improving and adapting myoelectric control systems (Pilarski et al., 2013a). This viewpoint is not surprising – in human action selection and decision making, situational information at both a low level (instantaneous information from sensory organs and afferent never fibers) and high level (e.g., cortical activity relating to location, emotional state, or long-term memory) is known to be integrated in multiple, complementary ways to modulate and enable action (Redish, 2013). In particular, knowledge encoded by learned predictions in the cerebellum can be influential in effecting timely and appropriate actions (Linden, 2003), and adaptable predictions made by the central nervous system seem to play an important role in human motor learning (Wolpert et al., 2001; Flanagan et al., 2003). Real-time machine learning of predictions and contextual information may be one way to provide the same kind of situation-appropriate modulation to sEMG controllers and other PMI devices (**Figure 4**).

A good starting point for situational or contextual awareness is the anticipation of human and robot dynamics, namely, predictions about changes in the stream of sensorimotor data flowing between the human, their device, and the device’s control system. As described in several recent studies, temporally extended prediction learning and anticipation can be made possible during the *ongoing* use of a human–prosthesis interface via techniques from reinforcement learning (Pilarski et al., 2012, 2013a; Edwards et al., 2013), namely the use of *nexting* with general value functions (Modayil et al., 2014). As described by Modayil et al. (2014), robot systems can now learn thousands of accurate predictions in a computationally efficient way from a single stream of data, in perpetuity, with learning and predicting occurring many times per second. Studies using computational *nexting* showed the ability to predict and anticipate the future position, motion, sEMG input signals, and contact forces of a myoelectrically controlled robotic limb (Pilarski et al., 2013a), to anticipate the control functions desired by a user (Pilarski et al., 2012), and also to predict the timing of a user’s control behavior (Edwards et al., 2013). This move towards more knowledgeable controllers supports and resonates with non-real-time PMI prediction learning work, e.g., the sEMG-driven predictions of upper-arm joint trajectories demonstrated by Pulliam et al. (2011). Creating systems that acquire and maintain predictive temporally extended knowledge regarding human–machine interaction has been shown to be both possible and potentially virtuous.

**FIGURE 5:**
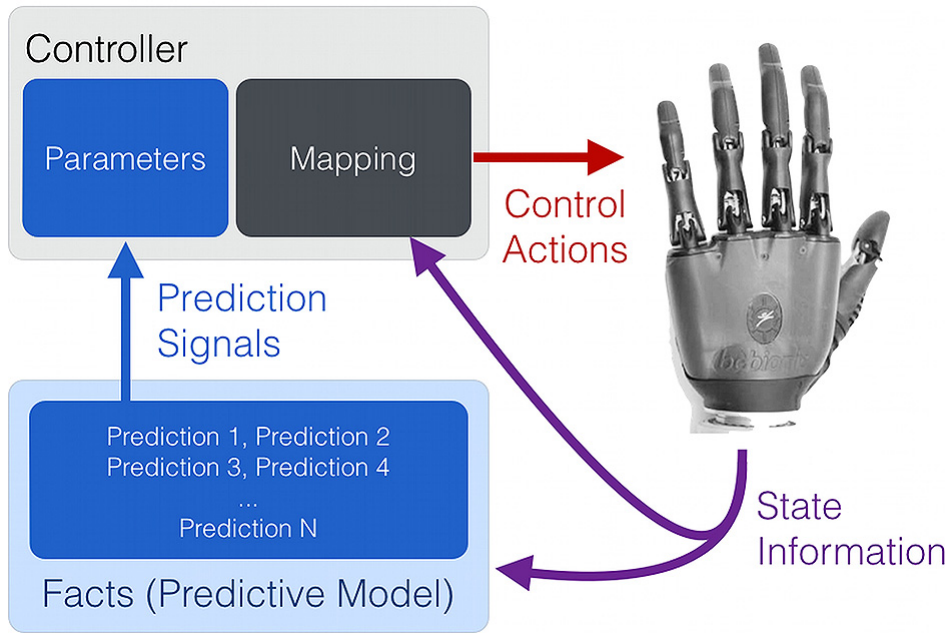
**Learned predictions can be used to adjust the control parameters of both conventional and emerging PMI controllers.** Examples include using situational predictions to dynamically re-order control options, change controller gains, or adapt thresholds and filters such that they are matched to a user’s immediate needs.

To be of benefit, situational sensorimotor knowledge must not only be learned in real time, but also deployed in real time to supplement the existing information available to a PMI device. As depicted in **Figure 5**, learned temporally extended predictions can be used to modify the internal parameters of conventional or pattern-recognition-based controllers during their ongoing use. Possible examples include using situational, user-specific predictions to dynamically re-order control options, change controller gains, or adapt thresholds and filters such that they are matched to a user’s immediate needs and physical condition. As suggested by Pilarski et al. (2012) and Edwards et al. (2013), a clear instance of this approach is the use of task- and user-specific predictions to optimize the control interface of a switching-based limb controller – e.g., dynamically change how co-contractions are interpreted to cycle through the numerous grip patterns of a dexterous hand prosthesis or sequentially controlled actuators of a robot limb. Once learned through active use, facts (predictions) about user preferences and past activity can be applied to rank-order control options in real time such that the correct options are made available to the user at the correct time (*dynamic* or *adaptive switching*, Pilarski et al., 2012).

**FIGURE 6:**
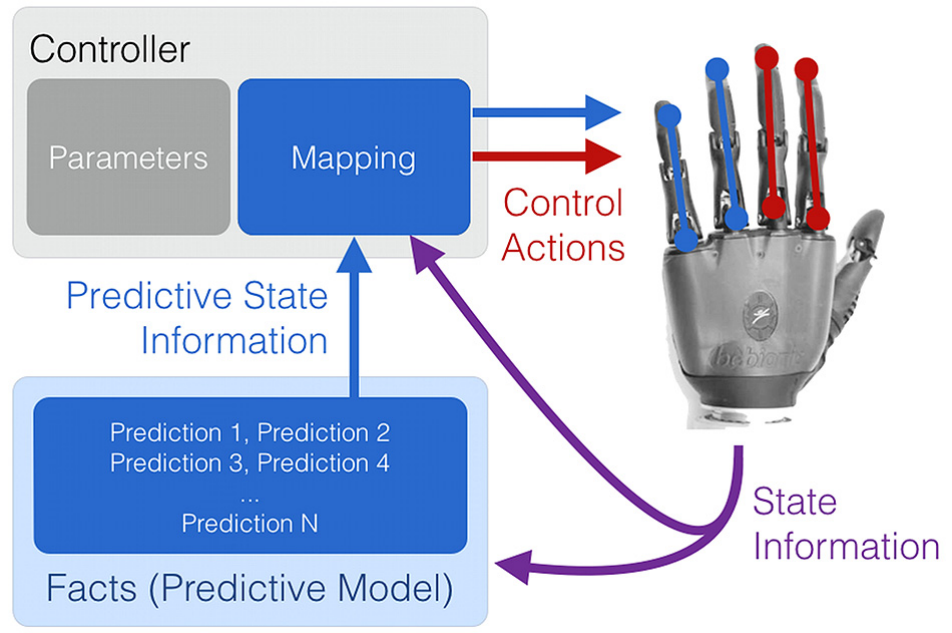
**Real-time machine learning provides up-to-date predictive state information to a control system.** Temporally extended predictions can serve as supplementary state information to improve control performance, or may be directly mapped to a set or subset of the available control functions.

Learned predictions may also be fed into controllers as additional state information (e.g., *predictive representations of state*; Littman et al., 2002). As depicted in **Figure 6**, this approach allows the flexible coupling of prediction learning with control, providing additional and perpetually up-to-date state information to a conventional or learned controller. Alternatively, predictions may be directly mapped in some way to predetermined movements, as is suggested to occur via the cerebellum and Pavlovian action selection in the brain (Linden, 2003; Redish, 2013). As one example, learned predictive state information has been shown to enable the simultaneous, anticipatory actuation of a supplementary wrist actuator during the myoelectric operation of other robot joints (Pilarski et al., 2013b).

In summary, learning and using situational sensorimotor knowledge appears to be a promising area for enhancing assistive devices, and there are preliminary results to show unsupervised adaptation, facilitation of simultaneous multi-joint control, and streamlining of interfaces that use switching. Using a real-time learning approach, predictions, and thus control behavior, can adapt during ongoing use without the need to explicitly redesign or retrain a controller. Real-time machine learning of predictions and anticipations may therefore present a way to preserve consistency in a control interface while at the same time allowing the control system to adapt quickly to things that are challenging (or impossible) for a designer to model prior to deployment. Continued work is this area will enable a move toward more advanced, persistent machine intelligence in PMIs and other assistive technologies.

## WHAT ALTERNATIVE, RADICALLY NEW SOLUTIONS ARE AVAILABLE, IF ANY?

Claudio Castellini, William Craelius, and Michael Wininger.

### OVERVIEW

Attempts to use sEMG signals for volitional control of advanced prostheses have not met with universal success, due in part to the fact that each central command propagates via variable transmission pathways to diverse muscles lying both superficially and deep, that act both synergistically and antagonistically, and in part to the fact that sEMG is labile to environmental factors endemic to the socket, i.e., moisture. The mechanical actions of the end-effectors, however, are better surrogates for volitions, since they embody trajectory, speed, and force directly, and are insensitive to upstream variability (Wininger et al., 2008; Yungher et al., 2011), and obviate the need for detection of neuroelectrical signals. Here we review two novel approaches for controlling a prosthesis based on measurement of the end-product of neural signaling, muscle activation.

### ULTRASOUND IMAGING

In the quest for novel peripheral interfaces, recently (Castellini and Passig, 2011; Zhou and Zheng, 2012; Guo et al., 2013; Sierra González and Castellini, 2013) medical ultrasound (US) imaging has proved its effectiveness as a means of detecting in real-time the position and force configuration of the human hand and wrist. Simple image processing techniques are applied to live ultrasound images gathered using standard medical ultrasonography devices. In the first case, the results have been used to control a simple, one-DOF wrist prosthesis, whereas in the second, anatomically irrespective features have been employed to reconstruct a subject’s desired metacarpo-phalangeal angles and fingertip forces. The reported accuracy results are in both cases comparable or superior to those obtained by sEMG, at the price of lower wearability and higher sensitivity to arm/hand displacement during the prediction.

In Sierra González and Castellini (2013) it is further shown that, in order to overcome at least the second drawback, a slightly more complex data gathering procedure could be used. As the learning/training procedure is extremely fast and simple, a wider sampling of the input space can potentially account for the inevitable movements of the subject’s arm and forearm. This seems a much simpler way than, e.g., to build a detailed model of the musculoskeletal structure, whose parameters should be assessed on a case-by-case basis given the inevitable inter-subject anatomical difference. Additionally, the learning system, based upon linear regression, is able to interpolate intermediate values from extreme values: it suffices to train on minimal and maximal forces/positions. This makes the approach realistic for usage with amputees.

All in all, from these results it seems that US has a future in the midterm run, but not as a direct competitor to sEMG. The main drawback remains the necessity of *carrying* the ultrasound transducer and the machine along; as it stands at the time of writing, the current technology forbids the complete miniaturization of such a device. Nevertheless, smaller and cheaper ultrasonographers are being built and marketed by the main manufacturers basically every year. On the other hand, more immediate applications are those in which the immensely richer information gathered from US imaging would really help. Firstly, wherever it is not strictly necessary to miniaturize the machine, although fine control is required; for instance, aboard a robotic wheelchair, or to control a robotic setup in the *domotic* framework (reaching, grasping, carrying). In such cases, related to patients with highly reduced mobility, US imaging could help, in that extremely tiny musculoskeletal changes could be fully detected and interpreted.

Secondly, US imaging could be used in a hospital as a day-care therapy for rehabilitation. Since every hospital in the Western world has nowadays access to ultrasound imaging (and the proposed approaches are irrespective of the characteristics of each single machine), it is imaginable to have muscle- and nerve-impaired patients, e.g., stroke and ALS patents, amputees, etc., attend periodic meetings to work out virtual-reality rehabilitation tasks. For instance, a virtual piano-playing application or the imitation of a visual model moving its arms and hands. The therapy could be finely tuned to each single patient. This therapy could be seen as a follow-up to mirror therapy (Ramachandran et al., 1995).

In both cases, and in particular if and when US-based control is required for a somewhat longer time than what is usually enforced in standard US examinations, it will be necessary to first investigate the effects of continuous ultrasound beams on the human tissues. US imaging is so far deemed harmless, but more testing in harder conditions is very likely to be required. Summing up, the usage of US imaging as a peripheral human–machine interface is being explored and is a promising alternative or complement to more portable – but less accurate – PMIs.

### TOPOGRAPHIC FORCE MAPPING

Signatures of the soft-tissue (i.e., mechanical) response of an upper-limb amputee can be represented as forces exerted by the entire residuum against the prosthetic socket. Volitions are thus encoded as topographic force maps (TFMs) that can be registered via a variety of pressure sensor arrays (Abboudi et al., 1999; Craelius, 2002; Yungher et al., 2011). Like ultrasound, this approach is not dependent on precise anatomical placement, and measures soft tissue response to neural activation.

Topographic force maps registers the 3D volume changes of the residuum are registered as a dynamic map of muscle recruitment. This measurement is made via force-sensitive resistors (FSRs), which change resistance according to force application normal to its sensor face. The FSR sensor comprises a resistive element printed onto a thin, flexible polymer, with a sensing head dimension as low as 5 mm diameter (3 mm active area). While the FSR material is not waterproof, the FSR’s manufacturer (Interlink, CA, USA) is ideally suited for placement within a waterproof enclosure.

Previous work with the FSRs in prosthetic sensing have placed them in a variety of arrangements: embedded in silicone or fabric, in direct- and indirect contact with the skin. In studies with generic sensor placement, the TFM technique shows high accuracy in predicting the basic movement parameters associated with isometric grasp, both in free- and targeted tasks (Wininger et al., 2008); this suggests feasible implementation as an off-the-shelf technology with self-application (donning and doffing). In clinical application of TFM, the sensors are more likely to be placed with an eye to capturing muscle activations at their most pronounced locations, i.e., bony prominences. In studies with custom sensor placement, outward pressures of the forelimb have shown high fidelity to basic grasp types and finger individuation (Craelius et al., 1999; Curcie et al., 2001; Phillips and Craelius, 2005). TFM-derived systems can be integrated into rehabilitation and training programs for retraining the upper-limb (Kuttuva et al., 2005; Kim et al., 2010; Yungher and Craelius, 2012).

Topographic force maps produce fast and accurate control over several independent DOFs by upper-limb amputees (Abboudi et al., 1999; Wininger et al., 2008; Yungher et al., 2011). Advantages of TFM over sEMG control include (1) better reliability due to inherently more reliable signals, (2) a resolution that is not dependent precision of sensor placement, allowing for convenient donning and doffing of the socket, (3) insensitivity to sweating, and (4) biomimetic, intuitive control. TFM-derived systems can be integrated into rehabilitation and training programs for retraining the upper-limb (Kuttuva et al., 2005; Kim et al., 2010; Yungher and Craelius, 2012). Recent work has extended the TFM paradigm to lower limb musculatures, showing high fidelity to basic parameters of the gait cycle in healthy ambulators (Yungher et al., 2011).

The practicality of TFM, and its limitations in terms of number of DOFs, and applicability to types of amputation, remains to be proven, since it has only been a laboratory technique thus far. Whether and to what extent this constrains the utility of TFM – and whether sEMG or any other detection modality has a greater likelihood of capturing these subtleties – remains to be seen: it may be that the enhanced stability of the TFM signal reduces the caprice as multifunction hands and wrists become more widely accessible (Scheme and Englehart, 2011). Clearly, TFM is limited by the dynamic mechanical environment within the socket, and in a way that other detection paradigms (including EMG and US) would not be. TFM has been studied only in “mature” residual limbs and primarily in patients with limb loss due to traumatic injury. In this way, TFM has been case-tested primarily in situations where there is relative stability in the residual limb shape and volume (Sanders and Fatone, 2011). In the future, testing TFM longitudinally and in patients with vascular disease would allow for important insight into how TFM performs across the stages of the recovery from amputation and in patients with potentially less stable volumetric change. However, we note that TFM does mitigate the issues associated with loss of signal baseline in re-application (i.e., it must be re-calibrated in doffing and re-donning), and classifiers built on the TFM calibration are likely to lose accuracy over time as the volumetric properties change within the residuum.

Topographic force map has not yet been tested in scenarios where grasp volition is to be decoded throughout a heavy lifting task. The socket and residuum are not rigidly connected, so placing a heavy weight in the prosthetic hand would shift the weight distribution across the surface of the limb, and would unload some sensors while increasing load on other sensors. This presents a unique signal decoding challenge not faced in EMG or US, and presents a need for further development in TFM.

## WHAT ARE THE BENEFITS OF SHARING CONTROL BETWEEN THE HUMAN SUBJECT AND THE PROSTHESIS?

Arash Ajoudani, Panagiotis Artemiadis, Antonio Bicchi, Strahinja Dosen, Dario Farina, Sasha Blue Godfrey, Mark Ison, Marko Marković.

### SEMI-AUTONOMOUS CONTROL OF UPPER-LIMB PROSTHESES

Recently, the prosthetic devices have evolved greatly, significantly growing in complexity from simple, single DOF grippers to highly dexterous systems providing individual finger control. However, the actual potential of these systems still remains largely under-utilized in daily life applications, as the current state-of-the-art PMIs cannot accommodate the emerging complexity of the system control. Conventionally, the development of PMIs has been driven mostly by the advances in the acquisition and processing of myoelectric signals, with the classical master–slave myocontrol being the most common command interface. In this traditional control setup, the prosthetic device (slave) “listens” for the muscle activity and then translates the user (master) intentions into actions. Here, we advocate a different approach that is based on developing systems which are capable of autonomous decisions making and independent, automatic operation, while at the same time sensing the environment and communicating with the user via a range of feedback interfaces.

**FIGURE 7:**
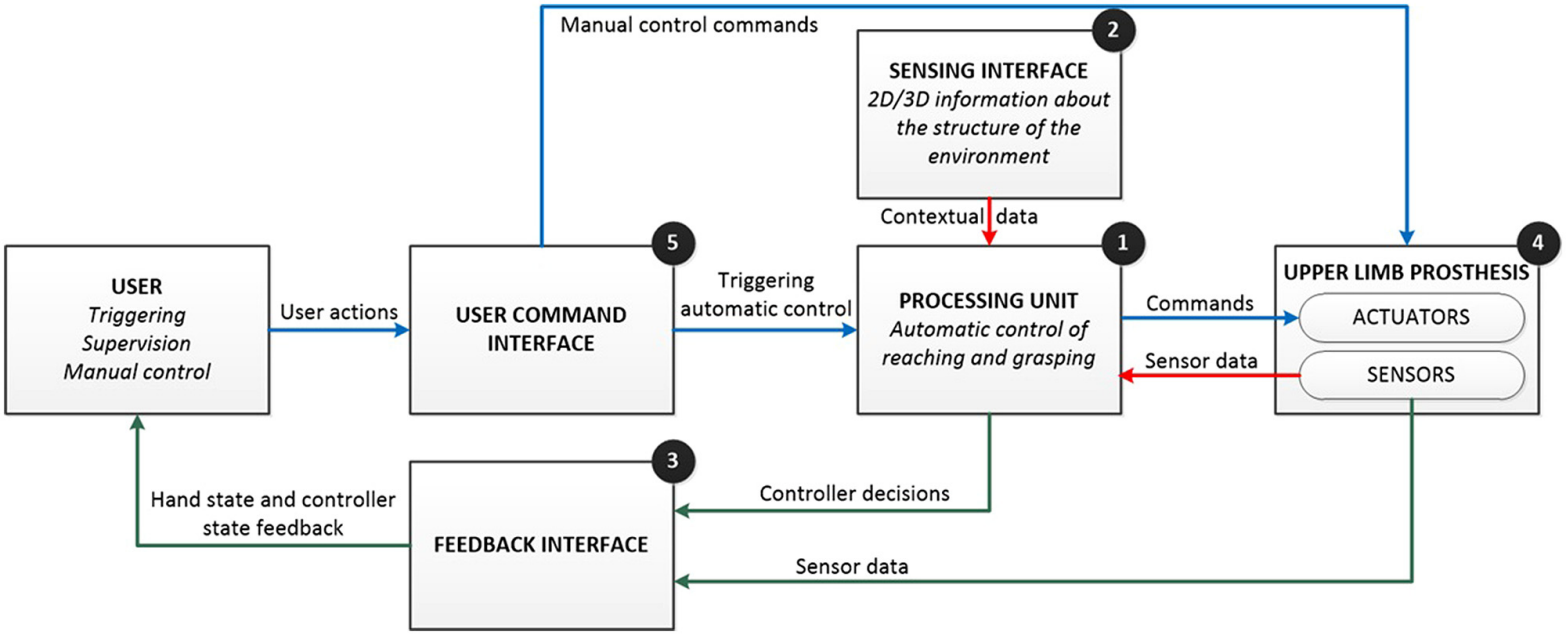
**Conceptual design of the semi-autonomous control of prostheses.** The basic idea is to enhance the artificial controller (processing unit) with an extra source of information (sensing interface) so that the system can operate automatically and autonomously, while the user has supervisory and corrective role. The main features of the system are automatic operation, bidirectional communication, semi-autonomous, and closed-loop control (see text for details). The flow of commands, sensor data, and feedback information are represented using blue, red, and green lines.

In its general form (**Figure 7**), such a system comprises: (1) a *processing unit (PU*; e.g., high-performance computing device) implementing control algorithms; (2) a *sensing interface* (e.g., cameras, inertial sensors) providing information for the autonomous decision making; (3) a *feedback interface* (e.g., vibrato- and/or electro-tactile stimulator, augmented reality display) communicating the state of the system to the user; (4) an upper limb prosthesis (e.g., a dexterous hand); and (5) a *user command interface* (e.g., myoelectric channels) providing high- and low-level manual control. The key features of the system are: *automatic operation*, *bidirectional communication*, *semi-autonomous*, and *closed-loop control*. Using a simple command interface, the user triggers the system operation. The PU acquires and analyzes the data from the sensing interface and then, independently from the user, commands the prosthesis to perform autonomous actions, such as, pre-shaping and orienting the hand to perform the grasp. Simultaneously, the PU uses the feedback interface to communicate the control decisions (e.g., selected preshape and orientation) as well as the current state of the prosthetic device (e.g., grasping force) to the user, thereby closing the control loop. The user can exploit this information to supervise the system operation and, when needed, take over the control to fine tune and/or correct online the automatic decisions of the artificial controller (bidirectional communication). Therefore, the control is shared between the user and the artificial controller, where the latter effectively shields the former from the low level execution details and thereby significantly decreases his/her cognitive burden (semi-autonomous control).

**FIGURE 8:**
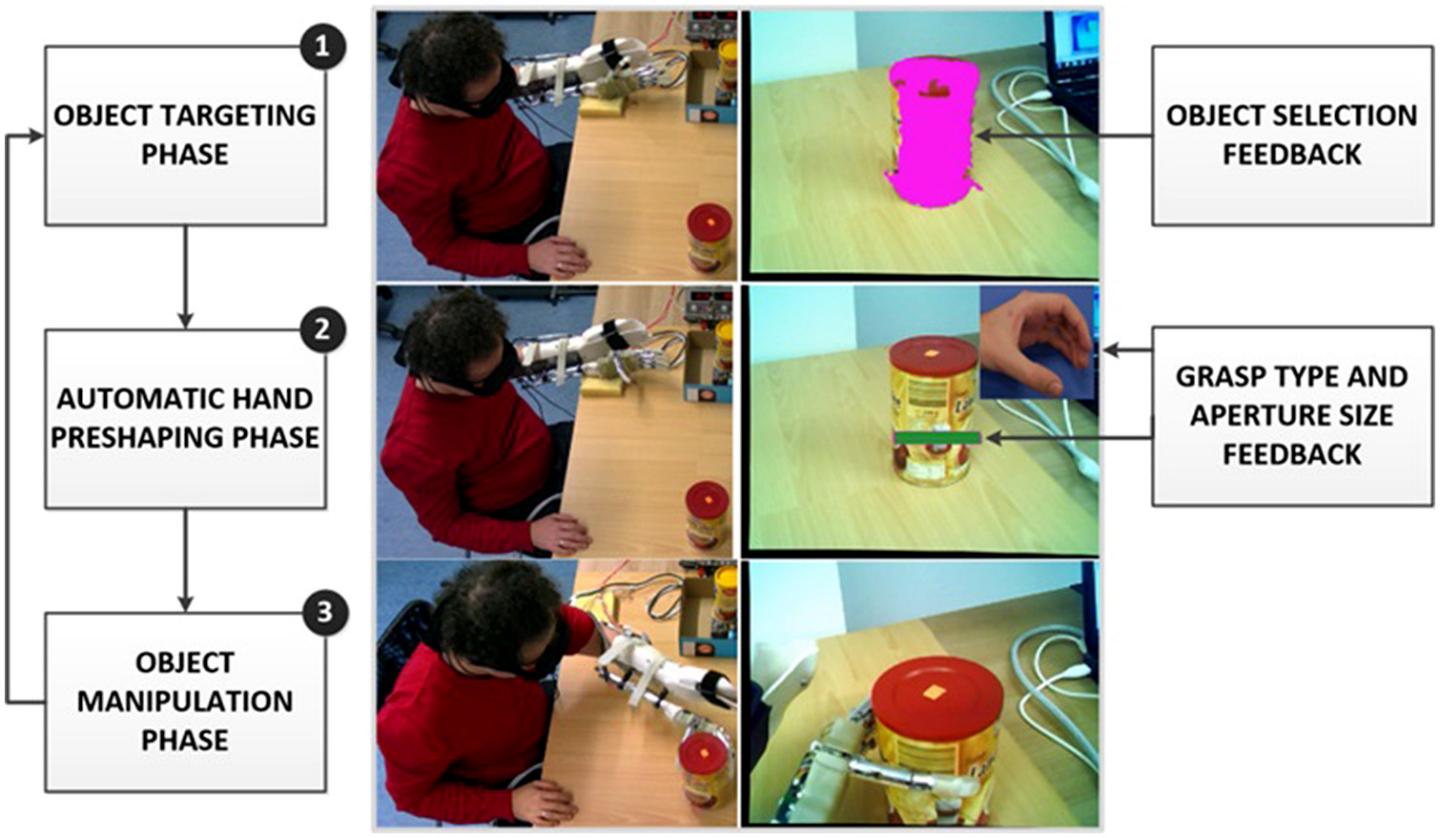
**Example operation of a prototype system implementing semi-autonomous control of grasping in a dexterous prosthetic hand.** The user wears augmented reality glasses equipped with a stereo camera pair and a stereoscopic “see-through” display. From top to bottom, the snapshots depict: (1) object targeting phase with augmented reality (AR) feedback about object selection, (2) automatic hand preshaping phase with AR feedback on the selected grasp type and aperture size, and (3) object manipulation phase. The panels on the right depict what the user actually sees through the glasses.

Representative examples of the above concepts have been presented in (Dosen et al., 2010; Dosen and Popović, 2011; Marković et al., 2013). In the prototype system for the control of grasping of a dexterous prosthetic hand, the user wears special glasses with embedded stereo cameras and an augmented-reality (AR) display. The glasses operate like a see-through interface, i.e., the cameras record the scene in front of the user, which is then projected stereoscopically to the display. The user triggers the operation of the semi-autonomous controller via a simple two-channel myoelectric interface. The system operation is organized as a state machine comprising several phases (**Figure 8**): (1) *object targeting:* the user looks at the object he/she would like to grasp. The computer vision is used to segment the scene and identify the targeted object. The system acknowledges the successful identification to the user by covering the object with a transparent overlay (i.e., AR feedback of the controller decision); (2)* automatic hand pre-shaping*: the user triggers the system indicating the intention to grasp the selected object. The controller determines the properties of the target object (shape and size), and based on this information, employs cognitive like processing (rule base) to decide grasp type and size suitable for the object. The hand is automatically preshaped and AR feedback communicates the selected grasp parameters to the user. The grasp type is shown as a visual icon in the peripheral visual field while the aperture size is depicted in the form of a virtual box placed next to the target object, where the size of the box corresponds to the amount of the hand opening; (3) *user corrections* the user evaluates the outcome of the automatic control (i.e., selected grasp type and size) by consulting the AR feedback and if needed adjusts the prosthesis preshape by issuing simple sEMG commands (bidirectional communication); (4) *object manipulation:* once the preshape is adjusted, the user issues the command for the prosthesis closing. The system was successfully evaluated in an experiment with 13 healthy subjects operating a prosthesis mounted on the forearm using a custom made splint (Marković et al., 2013). The full dexterity of the hand was utilized, i.e., the system controlled hand preshape by driving all available actuators to implement four grasp types and continuous range of aperture sizes.

The developed system is an illustrative example of how an artificial controller can be enriched with an additional, non-conventional information source (stereo camera pair), and a high level processing (cognitive-like reasoning) to achieve fully automatic control of the functions that are conventionally the responsibility of the user (e.g., hand pre-shaping; Jiang et al., 2012a). In this scheme, which is a form of the shared user-prosthesis control (see next section), the user is able to “release” predefined “motor programs” performing relatively complex functions, instead of continuously monitoring and controlling each step during the task execution. This substantially simplifies the myoelectric interface, which only needs to implement a simple triggering mechanism and also reduces the burden from the user. The presented control concept scales smoothly with the system complexity. For example, in the case of an entire upper limb prosthesis, the computer vision interface could be supplemented with inertial sensors tracking the prosthesis orientation in space. This could be used both to pre-shape the hand and to navigate the arm to reach and grasp the selected target object. Put differently, the complex “pre-shape and reach program” could be triggered via a simple myoelectric command. Finally, closing the loop through AR feedback has many potential advantages. Compared to the “classical” methods of tactile stimulation, AR feedback can utilize a much higher bandwidth of the visual communication channel.

The ultimate goal of this research is to make grasping and reaching using a complex dexterous hand and/or arm prostheses into a straightforward, routine activity, which corresponds to how these functions are performed in a daily life by able-bodied persons. Ideally, the subject would decide on the functional goal, and then he/she would simply trigger the system. The artificial controller takes over and autonomously implements all the low level details of the task execution. When delicate manipulation is necessary, however, the system allows the subject to assume a complete control of the system and fully focus on the task execution.

### SHARED USER-PROSTHESIS CONTROL

**FIGURE 9:**
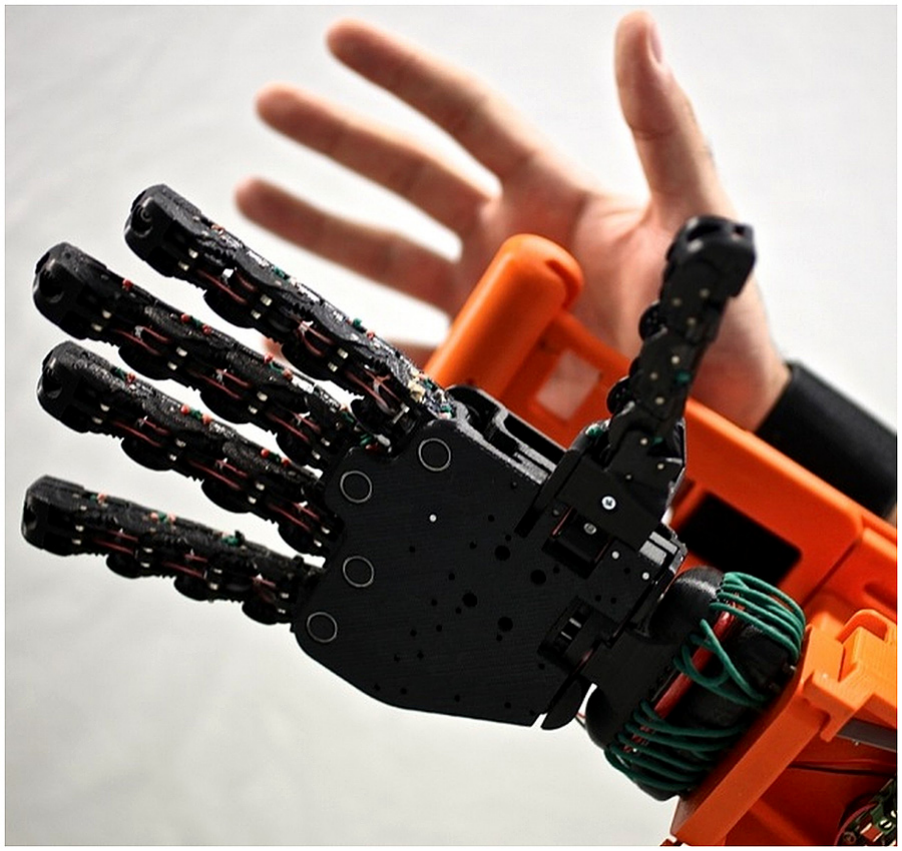
**The Pisa/IIT SoftHand and the forearm adapter used to test the device on control subjects**.

One approach to simplifying myoelectric interfaces is to share the burden of control between the hardware, software, and user. Many basic myoelectric devices make use of simple, proportional control but only allow one grasp with limited functionality. More complex systems require a different approach to control: current anthropomorphic hands require users to switch between postures in sequence before actually commanding the grasp, thus placing the burden almost entirely on the user. Pattern recognition and machine learning techniques, as are found in the literature (Naidu et al., 2008), in contrast, place that burden almost entirely on the software, which can require long training sessions and may limit the flexibility of the controller. By sharing control between the user and the device, one can achieve more malleable and intuitive control of complex systems. With the Pisa/IIT SoftHand (**Figure 9**; Catalano et al., 2014), we use a combined control strategy that takes advantage of the brain’s own means of simplifying hand movements to create an anthropomorphic hand with an intuitive control architecture.

The SoftHand design incorporates the motor control principle of synergies (Bernstein, 1967). With synergies, the brain is thought to command multiple DOFs, or joints, simultaneously in a coordinated pattern. Through principal component analysis (PCA; Santello et al., 1998) a mathematical representation of these synergies was developed and in turn used as the basis for a mechanical design (Bicchi et al., 2011). This design strategy was combined with principles from under-actuation (Birglen et al., 2008) to enable a soft robotics approach encode the movement pattern of the first PCA synergy. A single motor is thus used to pull a tendon that runs through the fingers and thumb. Because of the flexibility afforded by using a soft robotics approach, the SoftHand closes with a natural, human movement pattern that automatically molds around the object in contact. Additionally, the SoftHand fingers are designed to bend in virtually any direction in the event of a collision and then spring back to their original position, to avoid damage to the environment, hand, or human user.

Previous work on teleoperation of a robotic arm resulted in a shared control scheme dubbed teleimpedance (Ajoudani et al., 2011). This scheme uses sEMG to measure cocontraction levels of antagonist pairs to estimate joint stiffness. The goal was to increase transparency and intuitiveness of control by imbuing the robotic arm with human-guided stiffness modulation in addition to traditional position control. The teleimpedance algorithm developed for robotic tele-operation was modified and transferred to the SoftHand. Because the SoftHand contains only one motor, only one antagonist pair of muscles is needed to control the hand. To maximize the intuitiveness of the controller, the main external finger flexors and extensors were chosen as the controlling muscle pair, the *M. flexor digitorum superficialis* and the *M. extensor digitorum communis*, respectively. For higher level amputees, an alternative pair can be used, such as the biceps and triceps.

Establishing a complete biomechanical model of the forearm muscles to accurately map the sEMG signals of the sampled muscles to joint stiffness is impractical and likely invasive in an amputee population. For the SoftHand, two modified hyperbolic tangents were used in place of such a biomechanical model, one each to map the desired position and stiffness of the hand. To establish the parameters of the hyperbolic tangents, a brief calibration procedure is used in which the user repeatedly opens and closes the hand naturally (for position mapping) and at various self-selected levels of cocontraction (for stiffness mapping). In preliminary testing with five intact control subjects, normalized root-mean-square error rates of 17.6 and 13.4% were calculated for each synergy, respectively. Ultimately, parameter identification for these models in amputees is likely possible through various training methods such as mirror-box, teacher imitation, or mental imagery (Castellini et al., 2009).

**FIGURE 10:**
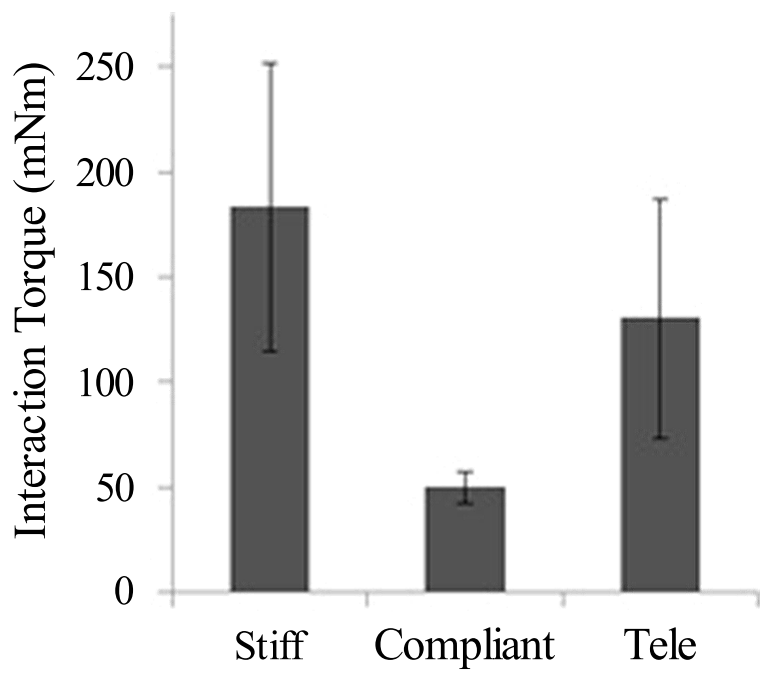
**Average interaction torques (in mNm units) by controller type (Ajoudani et al., 2014)**.

The teleimpedance controller described above was tested alongside stiff and compliant fixed-stiffness controllers with five intact subjects. Subjects were asked to grasp and lift objects of various size and weight off a table repeatedly; success rate and sEMG were recorded throughout the experiment. Interaction forces produced while using the teleimpedance controller were intermediate to those produced with the stiff and compliant fixed-stiffness controllers (**Figure 10**) and an intermediate ramp-up time was required to reach these forces (data not shown). The adaptability of the teleimpedance controller produced more favorable interactions with everyday objects: the stiff controller often resulted in object deformation when used to grasp more compliant objects, whereas grasps with the compliant controller were likely to slip despite adequate molding around the object. Another method to share control is to close the loop by providing feedback to the user. Because the human hand is capable of perceiving a variety of signals from temperature to surface texture to pressure, etc., in providing feedback to assist control, it is difficult to reproduce the full range of the hand’s sensory information. In preliminary testing, we have focused on feeding back grasp force to the user via vibrotactile motors (Godfrey et al., 2013). We have also explored mechanotactile force feedback and vibrotactile surface feedback. While still preliminary, feedback seems to enhance the user experience with the SoftHand and possibly limit fatigue effects, which is often a concern with prosthetic devices.

Results suggest the teleimpedance controller is a useful mechanism, potentially in combination with force feedback, to share the control burden. Ultimately, the proportion of the control the user and software/hardware are responsible for can shift to accommodate differing levels of needs and abilities. For example, for highly skilled users with the need for a greater variety of postures, more synergies can be incorporated, requiring a switching or selection from the user. Conversely, for users with minimal muscle control or limited musculature, the proportional force and teleimpedance control currently employed can be simplified to a simple on/off switch requiring only minimal signal from one muscle wherein the controller controls both the speed and stiffness of the hand.

### HUMAN-EMBEDDED CONTROLLERS FOR PROSTHETIC DEVICES

**FIGURE 11:**
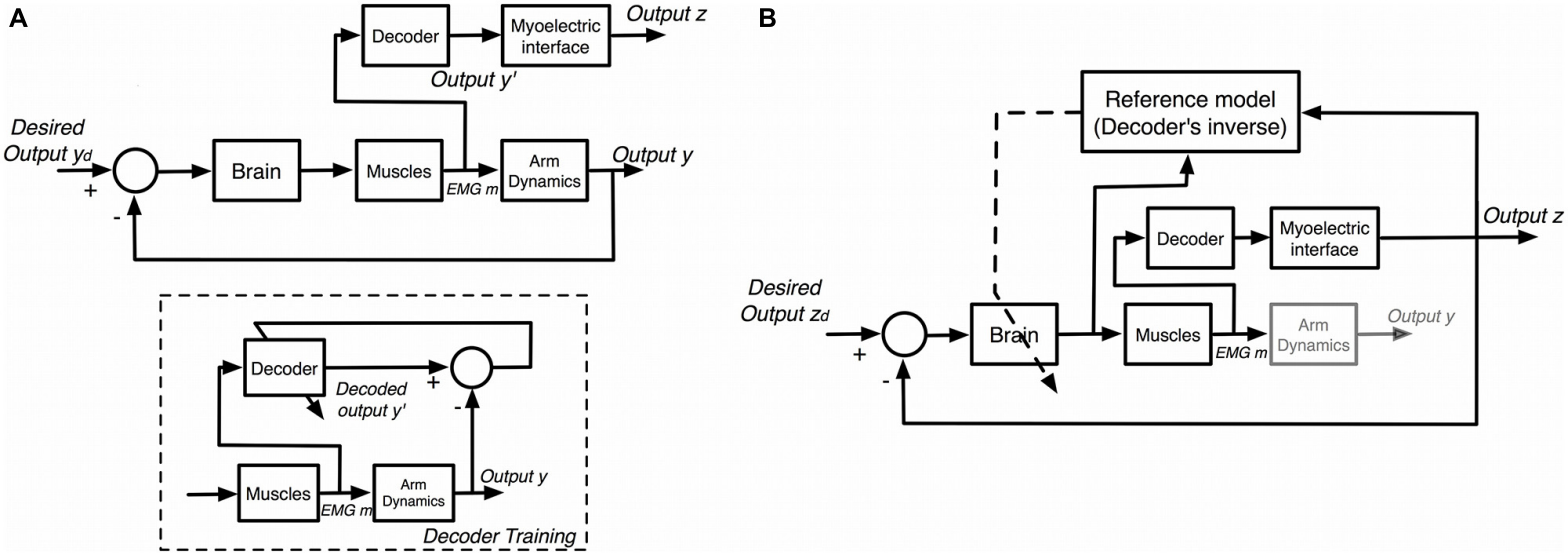
**General models of myoelectric interface interaction. (A)** Interfaces with trained decoders. A decoder is trained to map sEMG signals (m) to human arm motion (y). Once trained, the decoder is used in real-time to estimate arm motion (y′) and map it to output (z) for an interface. **(B)** Interfaces utilizing motor learning. The brain adjusts the neural commands based on the interface output (z) by learning the inverse model of the decoder.

With the desire for simultaneous and proportional control of multiple DOF prosthetic devices, recent research has stressed reducing the burden on users through intuitive controls that mimic human intentions (**Figure 11A**). However, placing the full burden on software prediction currently leads to more user frustration due to the aforementioned intensive training sets and limited prediction accuracy placing upper-bound constraints on user performance (Lorrain et al., 2011; Scheme and Englehart, 2013a). Alternatively, recent works have supported a shift in myoelectric control applications towards human-embedded controllers learned through interaction with a constant mapping function associating sEMG inputs with control outputs (Antuvan et al., 2014). Mussa-Ivaldi et al. (2011) propose that the human motor system is capable of learning novel inverse mappings relating the effect of motor commands on control outputs while interacting with myoelectric interfaces. This learning has been modeled and verified in the presence of closed-loop feedback (Radhakrishnan et al., 2008; Chase et al., 2009; Héliot et al., 2010), allowing users to perform tasks simply by learning controls in a given task space (Mosier et al., 2005; Liu and Scheidt, 2008; Liu et al., 2011; Pistohl et al., 2013). In this approach, the motor system adapts to the decoder, using knowledge of the inverse mapping to produce desired outputs, as depicted in **Figure 11B**.

Such controllers naturally integrate simultaneous and proportional controls through predefined mapping functions associating sEMG input with control outputs, and provide real-time learning that is difficult to achieve using pattern recognition techniques. This learning is prevalent for both intuitive (e.g., outputs roughly corresponding to limb motions) and non-intuitive (e.g., random mappings) mapping functions (Radhakrishnan et al., 2008; Antuvan et al., 2014). Although non-intuitive mappings are associated with a steeper learning curve, Antuvan et al. (2014) show they also incur higher learning rates capable of producing better performance over time compared to intuitive mappings, indicating that intuitive control schemes are not essential given the presence of feedback.

**FIGURE 12:**
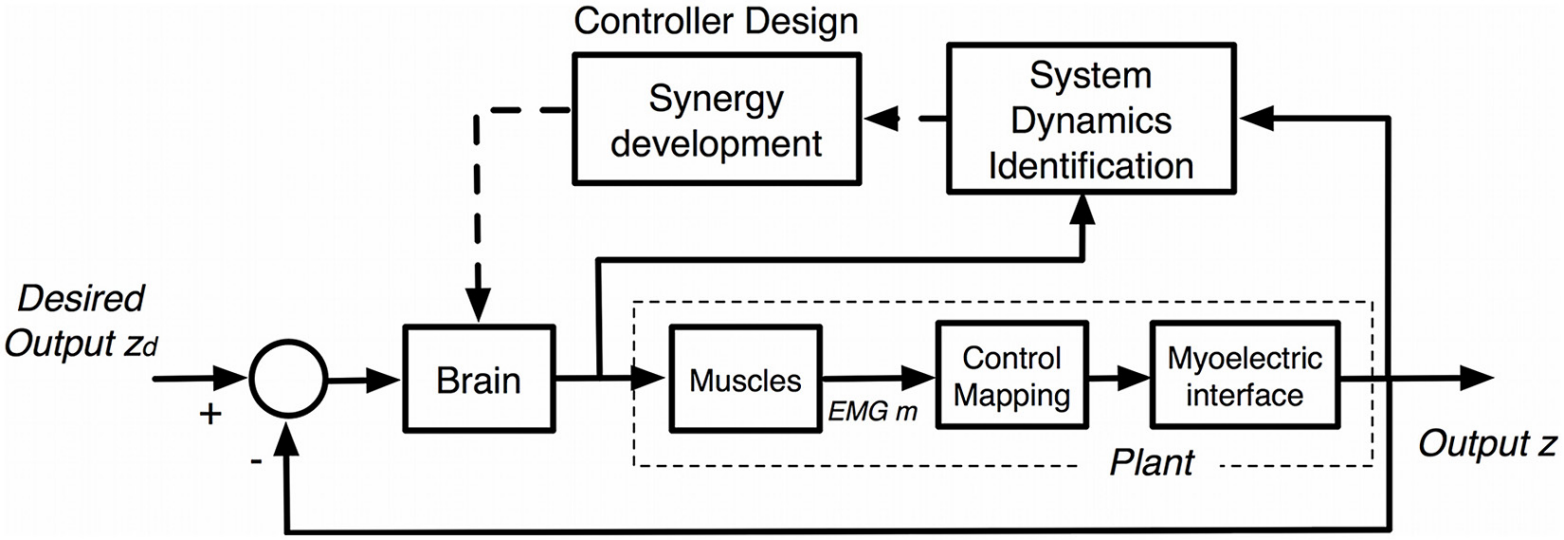
**Embedded brain control for myoelectric interfaces.** The brain learns a model of the plant to be controlled (system dynamics identification) by comparing neural commands and output (z) of the interface. New synergies are developed through controller design based on the system identified, which are then utilized while adjusting neural commands.

Recent work (Ison et al., 2014) has shown that users not only learn the mapping function relating sEMG with control outputs (**Figure 11**), but train their motor system to develop unique muscle synergies associated with the full system dynamics of the myoelectric device (**Figure 12**). Nazarpour et al. (2012) analyzed motor learning in the context of muscle synergies, which represent specific cross-muscle activation patterns used to achieve a behavioral goal (D’Avella et al., 2006). They set up a visual interface with common center to reach out tasks using cursor position control via pairs of biomechanically independent muscles. By examining user reactions to virtual perturbations in cursor position, they show that users obtain flexible control through the formation of dynamic, task-dependent muscle synergies.

Ison et al. (2014) recently analyzed long-term trends in human motor learning through interaction with similar visual interfaces incorporating human-embedded myoelectric controls. The work reveals the natural emergence of a new muscle synergy space as the user identifies the novel system dynamics of the interface (**Figure 12**). The system dynamics include not only the mapping function, but also disturbances from electrode placement and shift, limb position, and unique movement patterns, resulting in a robust control of the full task space. The developed synergies have common population-wide components, and their continuous refinement correlates with a long-term learning component that increases both performance and control efficiency over time during consistent, repeated use. Moreover, it is found that these synergies are maintained after periods of non-use, allowing subjects to retain a significant amount of performance on familiar tasks and generalize upon the introduction of new tasks within the same control space. The user then has freedom to increase control efficiency simply by interacting with the device to adaptively identify the system dynamics relating neural activity to the given, novel task space. The ability to retain and refine unique synergies and utilize them to generalize control to the entire task space supports the use of synergy development, not necessarily user-specific trained decoders, for efficient myoelectric control of robots designed for long term use.

Although these studies have only evaluated learning through visual interfaces, Pistohl et al. (2013) demonstrates the natural extension of human-embedded control to robotic devices. In addition, Ison et al. (2014) find that motor learning incurred using one interface translates to better initial performance with different interfaces utilizing the same mapping function, likely due to the use of the same previously developed muscle synergies.

Both studies suggest that users can be trained to control a multiple DOF myoelectric prosthetic device with minimal frustration, simply by interacting with the specific mapping function. In the case of amputees, especially TMR patients (Kuiken, 2006), there is opportunity to train the user to develop new synergies from residual muscles in order to achieve efficient control of a prosthetic device, robust to degradations currently plaguing pattern recognition methods. Thus, a shift in research focus toward human-embedded control potentially provides a novel and practical way to achieve user-friendly and robust myoelectric control of prosthetic devices.

## LESSONS LEARNED AND DISCUSSION

There is clearly no definite answer to any of the questions posed as the motivation for the workshop, but the opinions expressed in this paper are at least indicating a direction in which to go. Here is a list of recommendations for the future research in PMIs.

### APPROACH THE CLINICS

Research in PMIs is still essentially a matter of the academic community of rehabilitation robotics and machine learning; this means that the level of practical involvement of the scientists in the clinical environment is inadequate. The research focusses too much on the mathematics and the mechatronics and tends to neglect the final target, that is the patient. The clinicians involved in conceiving, designing, and fitting the prostheses and instructing the patients are still highly unsatisfied with the tools they get; this calls for a major change in the research perspective, which must be transferred on the field, namely, the hospital, or even at home, since the beginning.

A further theme which was not treated in the workshop is that of providing real-time feedback to the patient. So far, this branch of the PNS–MI topic, i.e., the feedback path, seems much less explored than its feedback counterpart. Nevertheless, the feedback path would definitely improve the feeling of embodiment, therefore strengthening the “reciprocal learning” effect, and overall enhancing the control. Subsequent editions of the workshop will take this issue into account.

### IMPROVE RELIABILITY

The tendency of machine–learning-based myoelectric control schemas to output unstable control signals is still a major issue, mostly caused by the inherent instability of biological signals. There are several suggestions to counter this problem. First and foremost, in the machine learning community there is still a dangerous tendency to claim that approach A is better than approach B on the basis that A achieves a better classification rate, whereas most of the times the classification rate is evaluated using the same dataset gathered oﬄine (Wagstaff, 2012), an idiosyncrasy denoted as “abstract versus concrete performance measures.” Concrete measures of performance involve tasks performed by the user in real life, e.g., how often a grasping action failed, how long it took to reach a target, etc.; these measures should become the gold standard. The community recommends that algorithms be first tested on standard benchmarks (which are still mostly unavailable) and then definitely tried in a clinical setup.

Secondly, machine–learning-based control is still, by and large, discrete and sequential, meaning that one DOF can be controlled on–off at each point in time (classification); as opposed to this, simultaneous and proportional control should be enforced. In this schema, one real-valued control signal is simultaneously available for each of the DOFs of the mechanical artifact. Each control signal should be independently controlled by the patient, possibly in the natural way (i.e., by “desiring” so), and short training should be enforced by devising a way to combine the single DOF activations into more complex patterns. It is clearly unacceptable to have the subject show the system, e.g., each and every grasping pattern.

Thirdly, novel PMIs should be used to improve the intent detection enforced by sEMG. There are indications that ultrasound imaging and topographic force mapping are viable approaches; computer vision for the estimation of the action to be taken, the development of new muscular synergies and the delegation of grasping to a lower-level closed-loop control are also interesting paths ahead.

### LACK OF EMBODIMENT

Most amputees will not feel that the prosthesis is their own hand, notwithstanding the well-known properties of adaptation shown by the human brain. This is due to at least two factors: lack of dexterity of the prosthetic devices (no prosthetic hand on the market allows for, e.g., force control of single fingers, let alone manipulation) and lack of feedback to the subject, let alone the need to improve the control, shorten the reaction times, and miniaturize the systems to be onboard. Ownership and immersion, the feeling of self with respect to the device, is in fact the ultimate goal.

## Conflict of Interest Statement

The authors declare that the research was conducted in the absence of any commercial or financial relationships that could be construed as a potential conflict of interest.
